# Implementing HIV teams sustainably improves HIV indicator condition testing rates in hospitals in the Netherlands: the #aware.hiv clinical trial

**DOI:** 10.1097/QAD.0000000000004167

**Published:** 2025-03-18

**Authors:** Carlijn C.E. Jordans, Klaske Vliegenthart-Jongbloed, Kara K. Osbak, Jaap L.J. Hanssen, Jan van Beek, Marion Vriesde, Natasja van Holten, Willemien Dorama, Dorien van der Sluis, Jurriaan de Steenwinkel, Jeroen van Kampen, Annelies Verbon, Anna H.E. Roukens, Casper Rokx

**Affiliations:** a Department of Medical Microbiology and Infectious Diseases; b Department of Internal Medicine, Erasmus University Medical Center; c Maasstad Hospital, Department of Medical Microbiology, Rotterdam; d Department of Infectious Diseases, Leiden University Medical Center; e Department of Viroscience, Erasmus University Medical Center, Rotterdam; f Department of Infectious Diseases, University Medical Center Utrecht, Utrecht, The Netherlands.

**Keywords:** healthcare practitioner, HIV, HIV indicator condition, HIV positivity rate, HIV testing, hospital, sexually transmitted infections

## Abstract

**Objective:**

Develop and validate a strategy to improve HIV testing rates using HIV teams.

**Design:**

A prospective clinical trial was conducted from January 2020 to July 2023 in two Dutch university hospitals.

**Methods:**

The intervention involved implementing HIV teams to provide peer awareness, education, and feedback to physicians treating patients ≥18 years newly diagnosed with HIV indicator conditions. The primary outcome was the HIV testing rate. Secondary outcomes included testing rates by specialty, HIV prevalence, and reasons for withholding testing.

**Results:**

Of the 313 666 newly registered diagnoses, 2395 involved indicator conditions. The overall HIV testing rate of newly diagnosed HIV indicator conditions increased from 50.1% (222/443) preimplementation to 80.7% (1575/1952) postimplementation of HIV teams (*P* < 0.001) with sustained improvement during the observation period (range 72.4–90.4%). The intervention was effective across physicians from all medical specialties. HIV prevalence among those tested was 0.6% [95% confidence interval (CI) 0.3–1.1]. Peer feedback for 411 untested indicator conditions, resulted in 69 (16.3%) additional HIV tests. Failure to test frequently remained without reason (50.6%) or due to patient loss of follow-up (18.4%). Multivariate analysis indicated that women with indicator conditions were tested less often [adjusted odds ratio (aOR) 0.59, 95% CI 0.45–0.79, *P* < 0.01], and indicator conditions without HIV testing recommendations in national guidelines were also less likely to be tested for HIV (aOR 0.36, 95% CI 0.27–0.48, *P* < 0.01). For external validation, we implemented this intervention in a second hospital, where it also significantly increased the testing rate postimplementation of HIV teams.

**Conclusion:**

Implementing HIV teams in hospitals is feasible, effective and leads to a sustained increase in HIV indicator condition-guided testing, supporting its broader adoption.

## Introduction

Over half of the people newly diagnosed with human immunodeficiency virus (HIV) in Europe are diagnosed during late stage HIV infection leading to more comorbidity and mortality [1–3]. HIV indicator condition-guided testing is an internationally recommended stigma-free strategy to improve timelier HIV case detection [4–6]. Indicator conditions share similar transmission routes as HIV or result from an immunocompromised state [5], and testing these conditions help healthcare providers to identify undiagnosed HIV in patients which guides medical management and prevents transmission [7].

A gap exists in the implementation of this testing strategy [7,8]. Indicator conditions are often missed by unaware healthcare providers [2]. This risk increases in patients who are older, women, migrant, or self-identify as heterosexual [2,9,10]. Consequently, people remain undiagnosed until they present with more advanced disease. Indicator conditions are present among all medical specialties making adequate clinical recognition by diverse healthcare providers instrumental to test for HIV. A lack of knowledge and a low HIV testing recommendation uptake in guidelines hinder this practice [11–13]. Implementing indicator condition testing has been possible in European hospitals [7]. Studies focused on a small selection of indicator conditions, one or few departments, and testing rates remained low. A hospital-wide proactive intervention to promote testing all HIV indicator conditions has not been performed.

We reasoned that direct medical specialist-led peer guidance substantially and sustainably increases HIV testing rates hospital-wide throughout medical specialties. Therefore, we pioneered by implementing HIV specialist-led HIV teams to ensure HIV indicator condition-guided testing in hospitals. Core tasks were to enable an active testing environment by peer-to-peer case audit and feedback with education of treating physicians. The study aim was to develop, test, and validate this methodology to improve HIV indicator condition-guided testing practices in hospitals.

## Methods

### Study design and participants

The #aware.hiv project is an ongoing prospective clinical trial at Erasmus University Medical Center (EMC), 1215 hospital beds, and expanded to Leiden University Medical Center (LUMC), 882 hospital beds, for external validation. Data collection started on January 1, 2020 in EMC and on April 1, 2022 in LUMC and lasted until July 31, 2023. This region has a relatively high HIV prevalence in the Netherlands [14]. Included were patients 18 years and older without HIV and a new HIV indicator condition diagnosis as defined by EuroTEST guidance (Appendix A, Supplemental Digital Content) [5,15]. Pregnancy and ‘conditions requiring aggressive immune-suppressive therapy’ were excluded because of, respectively, universal opt-out HIV testing and the subjectivity regarding aggressive immunosuppression which prevented efficient operationalization. People diagnosed with recurrent community acquired pneumonia were included, but a first occasion of pneumonia was excluded because of excess case load in a tertiary hospital setting. Lung cancer was excluded due to absent evidence as being HIV indicator condition in European settings [5,15] and significant physician testing hesitancy, unless another HIV risk factor was present. Cervical dysplasia was excluded in EMC only because a parallel nested sub study [16].

Patients with a negative HIV test in the year prior to the registered HIV indicator condition were considered adequately tested for HIV unless when mononucleosis-like illness or sexually transmitted infections (STIs) were diagnosed. Pregnant patients and patients until one year postpartum were considered adequately tested due to universal pregnancy screening [17]. HIV team intervention in EMC started later for dermatology and neurology departments due to the medical team's preference to have educational session first.

### Intervention

The intervention was the implementation of HIV teams, which consisted of HIV medical specialists (registered internist-infectiologist, and a medical microbiologist if available) and nurses, and data collectors (master or PhD students, residents, or HIV specialized nurses), starting August 1, 2020 in EMC and October 1, 2022 in LUMC. Six postimplementation periods were defined from August 1, 2020 until end of data collection: postimplementation period 1 (August 1, 2020–December 31, 2020), postimplementation period 2 (January 1, 2021–June 30, 2021), postimplementation period 3 (July 1, 2021–December 31, 2021), postimplementation period 4 (January 1, 2022–June 30, 2022), postimplementation period 5 (July 1, 2022 – December 31, 2022), and postimplementation period 6 (January 1, 2023–May 21, 2023).

**Fig. 1:**
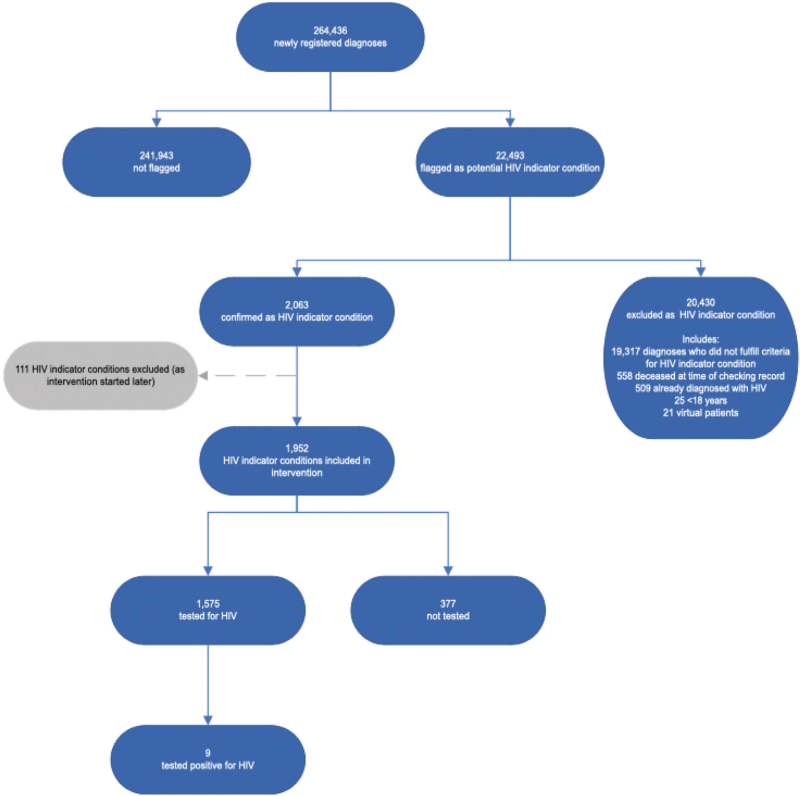
Flowchart of HIV indicator conditions at EMC postimplementation of HIV teams.

The HIV team operated hospital-wide and used peer-to-peer feedback on a per case basis and education. All peer-to-peer feedback was given either verbally or via E-Mail with contact details for further questions. All departments were informed on project at the start of first implementation period. Treating physicians of patients newly diagnosed with HIV indicator conditions untested for HIV received HIV testing recommendations by direct contact and by notes in the electronic health record. The HIV teams also organized department-adapted educational sessions and yearly hospital HIV awareness events. In EMC we piloted the effect of adding HIV testing recommendations to the local guideline for cervical cancer (per December 1^st^, 2020) and implemented information communication technology (ICT)-integrated prompts (per December 2022) (Figure 1, Supplemental Digital Content) with automated textual HIV testing recommendation added to specific microbiological diagnostics test results indicating HIV indicators (Appendix B, Supplemental Digital Content).

### Data collection

We developed a semi-automatic tool that used International Classification of Diseases, tenth revision (ICD-10) and standardized health insurance (DBC) codes to flag potential HIV indicator conditions in the hospital electronic health records system. A list of all newly registered potential HIV indicator conditions was generated biweekly. Data collectors screened electronic health records to verify indicator conditions, excluded conditions with alternative explanations, and checked available HIV testing. Unclassifiable cases were discussed within the HIV team. Clinical definitions (Appendix A, Supplemental Digital Content) could be updated in this pilot phase based on these discussions to better specify included conditions. Pre and postimplementation data were collected on patients with HIV indicator conditions and testing.

### Outcomes

The primary outcome was the overall HIV testing rate of newly registered HIV indicator conditions postimplementation of HIV teams, compared to preimplementation. Secondary outcomes were HIV testing rates per medical specialty and per HIV indicator condition. We also assessed testing rates stratified by patient sex, age, time-period, and HIV testing recommendations uptake in national specialty guidelines as well as reasons provided to withhold HIV testing, ICT-integrated diagnostics testing yield, and HIV prevalence.

### Statistical analysis

HIV testing rates were assessed by calculating the proportion with Wilson 95% confidence interval (CI) of diagnosed HIV indicator conditions tested for HIV relative to the total number of diagnosed HIV indicator conditions. The HIV prevalence with CI was assessed using the proportion of HIV indicator conditions that tested positive for HIV relative to the total number of tested HIV indicator conditions. To compare HIV testing rates, chi-square tests were used. A Wald chi-square multivariate logistic regression analysis was performed to evaluate factors influencing HIV testing, including patient age and sex, the specialty registering the indicator condition, and the presence of HIV testing recommendations in national specialty guidelines. The reference levels in the categorical variables were chosen to obtain clinical relevant comparisons.

### Ethical statement

The study procedures were carried out in accordance with the regulations of the Declaration of Helsinki. Approval of the research Medical Ethics Committee, Erasmus University Medical Center, Rotterdam, was obtained (MEC-2020-0140). The Medical Ethics Committee waived the need for individual informed consent with the use of data from routine care.

## Results

From January 1, 2020, a total of 313 666 diagnoses were newly registered at EMC (total diagnoses newly registered during the study period), including 49 230 preimplementation and 264 436 postimplementation of HIV teams. Among these, 26 653 (4160 preimplementation and 22 493 postimplementation) (8.5%, 95% CI 8.4–8.6) were flagged as potential HIV indicator conditions. In total 2506 (443 preimplementation and 2063 postimplementation) (9.4%, 95% CI 9.1–9.8) were confirmed as HIV indicator conditions after manual screening, resulting in an overall HIV indicator condition prevalence of 0.80% (95% CI 0.77–0.83). Following the exclusion of 111 diagnoses due to the later start of HIV team interventions for three specific conditions (cervical cancer, atypical or severe psoriasis, and seborrheic dermatitis/exanthema), 2395 diagnoses, of which 1952 postimplementation, were included in the primary outcome analysis (Fig. 1). The median age of patients with HIV indicator conditions postimplementation of HIV teams was 51 years (interquartile range, IQR 38–75 years) with 52.3% (1020/1952) of the diagnoses occurring in women.

HIV, human immunodeficiency virus. ∗ Virtual patients: patients who have only been discussed in multidisciplinary consultations but have never physically been in the hospital.

### Prevalence of HIV indicator conditions

The most prevalent registered HIV indicator conditions postimplementation of HIV teams were cervical cancer (381/1952, 19.5%), non-Hodgkin lymphoma (272/1952, 13.9%), and STIs (193/1952, 9.9%). The internal medicine department diagnosed most HIV indicator conditions (772/1952, 39.5%), followed by gynecology/obstetrics (249/1952, 12.8%), and dermatology/venereology (243/1952, 12.4%). The prevalence of individual HIV indicator conditions from the preimplementation period was comparable to the postimplementation period (Table 1, Supplemental Digital Content).

### HIV testing rates over time

**Fig. 2:**
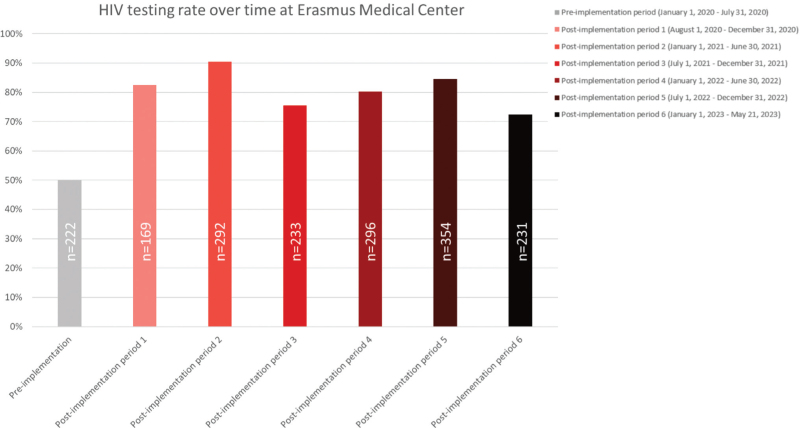
Overall HIV testing rate over time, pre and postimplementation of HIV teams.

The overall HIV testing rate of HIV indicator conditions significantly increased from 50.1% (222/443) preimplementation to 80.7% (1575/1952) postimplementation of HIV teams (*P* < 0.001). The overall HIV testing rate showed a sustained increase with variability over time (range 72.4–90.4%) (Fig. 2).

### HIV testing rates per specialty

**Fig. 3:**
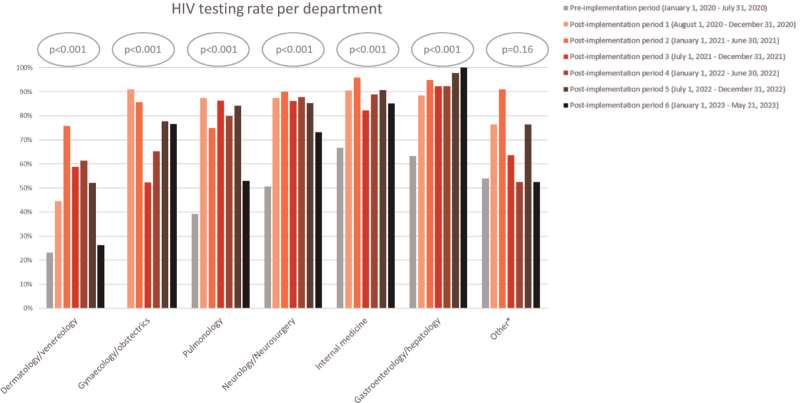
HIV testing rates over time per specialty, pre- and postimplementation of HIV teams.

When assessing the HIV testing rate per specialty postimplementation over time, all increased initially and a sustained increase was found among all main specialties, except for dermatology/venereology (Fig. 3) The specialties gastroenterology/hepatology, internal medicine and neurology/neurosurgery showed the highest overall HIV testing rates postimplementation of HIV teams, 95.5% (189/198), 88.9% (686/772), and 85.0% (214/252) respectively. The lower overall testing rates of HIV indicator conditions within dermatology/venereology were driven by the testing rates for severe or atypical psoriasis (22/62, 35.5%), seborrheic dermatitis/exanthema (5/11, 45.5%), and herpes zoster (9/18, 50.0%) (Table 3, Supplemental Digital Content). The declining trend observed in postimplementation period 6 resulted from progressively lower testing rates for severe or atypical psoriasis (2/15, 13.3%) and STIs (5/19, 26.3%). The testing rate found within gastroenterology/hepatology was mostly driven by universal or near universal testing rates for hepatitis A, B, and C after HIV team implementation (3/3, 100%, 132/136, 97.1%, and 44/45, 97.8%, respectively). The highest absolute increase in testing rate was observed for cervical cancer (0/44, 0% preimplementation to 279/381 73.2% postimplementation, *P* < 0.001) where the implemented HIV team coincided with a local guideline adaptation. Furthermore, the ‘other’ category showed a substantial variation in HIV testing rates over time as a result of the number of departments (*n* = 8) included in this group yielding low numbers (mean 21) of HIV indicator conditions per time period.

HIV, human immunodeficiency virus. ∗ Other included the following departments: cardiothoracic surgery, ophthalmology, orthopedic surgery, otorhinolaryngology, psychiatry, rheumatology, surgery, and urology. *P*-value indicates the comparison between the mean proportion of HIV indicator conditions per specialty tested for HIV preimplementation vs. the mean proportion of HIV indicator conditions per specialty tested for HIV postimplementation calculated using chi-square tests

### HIV testing rates per indicator condition

AIDS defining conditions and non-AIDS defining conditions showed comparable HIV testing rates postimplementation (82.5% vs. 79.5%, *P* = 0.1). Considering testing rates per HIV indicator condition, we found that four conditions (unexplained leukocytopenia/thrombocytopenia (44/44), unexplained fever (25/25), multiple sclerosis-like disease (9/9), and recurrent pneumonia (9/9)) had a 100% testing rate postimplementation of HIV teams (preimplementation 78.6% (11/14), 92.9% (13/14), 50.0% (2/4), and 50.0% (2/4), respectively). The following conditions reached testing rates above 90% postimplementation of HIV teams: non-Hodgkin lymphoma (from 92.7% (38/41) preimplementation to 96.0% (261/272) postimplementation), Kaposi's sarcoma [from 66.7% (4/6) to 92.9% (13/14)], malignant lymphoma [from 75.0% (3/4) to 98.5% (66/67)], hepatitis C [from 80.0% (12/15) to 97.9% (47/48)], hepatitis B [from 81.8% (27/33) to 96.6% (142/147)], unexplained chronic renal impairment [from 61.5% (16/26) to 95.6% (65/68)], lymphocytic meningitis [from 77.8% (7/9) to 94.0% (47/50)], unexplained weight loss [from 86.7% (13/15) to 93.1% (54/58)], and idiopathic thrombocytopenic purpura [from 100.0% (3/3) to 92.6% (25/28)]. The lowest HIV testing rates were found for the indicator condition Guillain-Barre syndrome [from 33.3% (2/6) to 27.3% (3/11)], severe or atypical psoriasis [from 28.6% (2/7) to 36.4% (28/77)] and lung cancer where the presence of an HIV risk factor was known [from 0.0% (0/10) to 7.7% (1/13)] (Table 1, Supplemental Digital Content). Further exploring the test rates of STIs, the overall test rate more than doubled in this population (from 30.0% (12/40) preimplementation to 62.7% (121/193) postimplementation). Syphilis had the highest testing rate [from 80.0% (4/5) preimplementation to 95.5% (42/44) postimplementation], whereas testing rates for other STIs were considerably lower (Table 2, Supplemental Digital Content).

### Factors associated with indicator conditions being tested for HIV

The multivariate analysis indicated that women (adjusted odds ratio, aOR 0.59, 95% CI 0.45–0.79, *P* < 0.01) and patients with indicator conditions without HIV testing recommendation in national treatment guidelines (aOR 0.36, 95% CI 0.27–0.48, *P* < 0.01) were less often tested for HIV (Table 1). Age was not associated with indicator conditions being tested for HIV (*P* = 0.25).

**Table 1:** Logistic regression multivariate analysis of factors associated with indicator conditions being tested for HIV.

Factor	aOR	95% CI	*P*-value
Age (years)			
≤50	0.86	0.67–1.11	0.25
>50 (ref.)			
Sex			
Female	0.59	0.45–0.79	<0.01^∗^
Male (ref.)			
Departments			
Dermatology/venereology	0.07	0.03–0.13	<0.01^∗^
Gynaecology/obstetrics	0.40	0.18–0.86	0.02^∗^
Pulmonology	0.20	0.09–0.46	<0.01^∗^
Neurology/neurosurgery	0.43	0.20–0.95	0.04^∗^
Internal medicine	0.58	0.27–1.15	0.12
Other^a^ Gastroenterology/hepatology (ref.)	0.15	0.07–0.33	<0.01^∗^
HIV testing recommendation in national guideline			
No	0.36	0.27–0.48	<0.01^∗^
No guideline available	1.27	0.77–2.11	0.35
Yes (ref.)			

aOR, adjusted odds ratio; CI, confidence interval; HIV, human immunodeficiency virus.

∗ Significant (*P* ≤ 0.05).

a Other included the following departments: cardiothoracic surgery, ophtalmology, ortopedic surgery, otorhinolaryngology, psychiatrics, reumatology, surgery, and urology.

### Peer-to-peer feedback

Postimplementation of HIV teams, healthcare providers adequately tested 1506 HIV indicator conditions. The HIV team identified 446 untested cases and gave peer-to-peer feedback in 411 cases, leading to 69 (16.3%) extra HIV indicator conditions being tested for HIV. Reasons for the 35 cases where no peer-to-peer feedback was given were: missed by the HIV team (*n* = 23), had HIV testing planned but not performed (*n* = 7), concerned terminal care patients (*n* = 3), or the test offer was refused by the patient (*n* = 1) or doctor (*n* = 1). HIV testing recommendations were most often given for the indicator conditions cervical cancer, STIs, and psoriasis with HIV test uptake rates of 13.4% (15/112), 19.5% (16/82), and 12.7% (7/55) respectively.

Out of 377 untested HIV indicator conditions (Fig. 2), 342 received HIV testing recommendations without uptake of HIV testing. In half of the cases (173/342, 50.6%), no reason to withhold HIV testing was provided (Tables 4 and 5, Supplemental Digital Content). Most common reasons provided not to test for HIV were the patient not returning to the hospital after HIV testing advice (63/342, 18.4%), the HIV test was included in the diagnostic plan after advice, but eventually not performed (35/342, 10.2%), and absent clinical indication to test for HIV according to the treating physician (29/342, 8.5%). Notably, only 4 (1.2%) patients did not accept the HIV test after being offered by the physician. STIs frequently had no medical follow-up which prevented HIV test uptake (15/66, 22.7%) and in 16.7% (11/66) of STI cases the physician advised HIV testing in the routine care for STIs in the Netherlands (general practitioner or at a sexual health clinic).

### ICT-integrated prompts for HIV testing

From December 2022 onward, physicians received testing prompts for a total of 580 samples from 420 patients by the ICT-integrated diagnostics tool. Of these, 95 (22.6%) patients were already known to be HIV positive and 158 (37.6%) patients were diagnosed with an HIV indicator condition. Most diagnosed HIV indicator conditions were invasive pneumococcal disease (72/158, 45.6%), chlamydia (31/158, 19.6%), syphilis (26/158, 16.5%), and *Mycobacterium tuberculosis* (18/158, 11.4%). The overall HIV testing rate increased significantly from 60.3% (120/199 patients) between August 2020 until implementation of ICT-integrated prompts to 73.4% (116/158) after implementing ICT-integrated prompts (*P* = 0.01) with most effect on invasive pneumococcal disease [from (25/64) 39.1% to (51/72) 70.8%] (Table 6, Supplemental Digital Content).

### HIV positivity rate

Postimplementation of HIV teams, nine (0.6%, 95% CI 0.3–1.1) HIV indicator conditions tested positive for HIV compared to one in the preimplementation period (0.5%, 95% CI 0.4–2.3%, *P* = 0.43). The ten registered HIV indicator conditions were diagnosed in seven patients aged between 34 and 65 years. Most were male and presented with an AIDS defining condition (Table 2).

**Table 2:** Characteristics of patients who tested positive for HIV.

Patient	Phase	Sex	Age	HIV indicator condition	Department(s) that registered diagnosis	Viral load (copies/mL)	CD4^+^ T-cell count (cells/mm^3^)	CDC classification	HIV transmission route
1	Preimplementation	Male	52	Non-Hodgkin lymphoma	Internal medicine	5.00E5	110	C3	MSM
2	Postimplementation phase 1	Female	65	Cerebral toxoplasmosis	Neuro-surgery	7.99E5	20	C3	Hetero-sexual contact
3	Postimplementation phase 2	Male	34	Syphilis	Internal medicine and ophthalmo-logy	2.22E5	290	B2	Hetero-sexual contact
4	Postimplementation phase 2	Male	50	Unexplained fever	Internal medicine	3.26E6	90	B3	MSM
5	Postimplementation phase 4	Male	37	Primary space occupying lesion of the brain – Cerebral toxoplasmosis	Neuro-surgery and neurology	1.46E5	20	C3	Unknown
6	Postimplementation phase 4	Male	50	Cerebral toxoplasmosis	Neuro-surgery and internal medicine	6.85E5	40	C3	Unknown
7	Postimplementation phase 5	Female	53	Hepatitis B	Gastro-enterology	7.14E4	40	B3	Hetero-sexual contact

a https://www.aca.gov.hk/publication/g40.pdf. MSM, men who have sex with men.

### External validation of the implementation of HIV teams

From October 1, 2022 onward, a total of 68 441 diagnoses were newly registered at LUMC. Of these, 2075 (3.0%) were flagged as potential HIV indicator conditions. After manual screening, 172 HIV indicator conditions (8.3%, 95% CI 7.1–9.6) were confirmed (Supplementary Figure 2). Most HIV indicator conditions were diagnosed in females (101/172, 58.7%), with a median age of 53 years (IQR 39–67). The HIV testing rate in LUMC increased from 51.8% (87/168) preimplementation of HIV teams to 66.3% (114/172) after implementation of HIV teams (*P* < 0.01). The HIV positivity rate postimplementation was 0.9% (95% CI 0.0–4.1) and was not significantly different from the HIV positivity rate preimplementation (0.0% 95% CI 0.0–2.2, *P* = 0.22).

## Discussion

We designed, successfully implemented, and externally validated the effectiveness of HIV specialist-led HIV teams in hospitals to increase HIV testing in patients with HIV indicator conditions. This targeted approach improved HIV testing practices of healthcare providers in hospitals substantially and sustainably. Improvements were observed across medical specialties and HIV indicator conditions. The data point towards a generally more willing testing atmosphere in hospitals and confirm our hypothesized improvement on HIV indicator condition-guided testing by putting HIV specialists in a central and proactive role among peers and the diagnostics trajectories of their patients.

The observed increase in the HIV testing rates was higher than a related strategy recently described in a similar setting but differing on one important aspect [18]. Our strategy coupled education and audits to individual peer-to-peer feedback instead of feedback on medical specialty level. Such personal approach of audit and feedback could well have driven the observed difference [19]. The testing rates achieved with our hospital-wide intervention matched the rates of interventions focusing on one particular indicator condition or department [7,20], and exceeded prior studies in Europe on selected conditions and departments [21–23]. The observed HIV prevalence was higher than the 0.1% cost-effectiveness threshold, as expected based on the prevalence in prior studies [18,22,24–26].

### Missed opportunities

Still around 25% of HIV indicator conditions remained untested. The specialties gastroenterology/hepatology, internal medicine, and neurology/neurosurgery performed best, while dermatology/venereology did poorer, contrasting previous studies [21,25]. Although not studied here, a perception of testing futility among peer groups of medical specialists may lower audit and feedback effect but this hypothesis needs exploration [5]. Patient refusal was not a barrier, in line with prior literature [18]. Understanding reasons why individual physicians withhold HIV testing may help to improve interventions. Recent publications from others and us indicate a prominent stigma among healthcare providers in hospitals, underscoring the potential relevance of behavioral interventions [27,28]. A second reason to withhold testing in hospitals is practical as some conditions, for example STIs in the Netherlands, are generally tested in first-line healthcare.

### Drivers of the HIV testing rate

Guidelines with HIV testing recommendations increased testing rates. The frequent omission of HIV testing recommendations in guidelines described in prior work [11], warrants urgent attention. Guideline uptake can overcome physician skepticism and address low HIV testing rates in specific groups at risk to remain untested such as women. Leveraging ICT-integrated prompts can also promote testing. A higher exposure to patients with HIV indicator conditions led to more sustained testing rates in medical specialties, possibly indicating a relevant learning effect from repetitive exposure.

### Strengths

As first study, we comprehensively included all conditions listed by the EuroTEST guidance with an intervention acting at all hospital departments. Second, the study follow-up was longer than prior studies on indicator condition-guided testing. Third, the external validation strengthens the possible generalizability of the results. Last, the intervention proved adaptable, offering flexibility to tailor the approach to local contexts depending on staff availability for education and indicator condition screening, to prioritize specific HIV indicator conditions or departments, and utilize available ICT infrastructure for electronic prompts or automated screening. The current data support that HIV teams can improve testing rates, and consequently HIV care, in a similar way to antibiotic stewardship teams (A-teams) which have improved the use of antimicrobials [29,30]. This all supports implementing HIV teams in other settings internationally.

### Limitations

First, we cannot exclude physician ICD-10 registration leading to miscoding or delays, although its mandatory use for financial management mitigated this risk. Second, inter-observer variability may have affected data collection, though standardized operating procedures and regular meetings addressed this. Third, screening for HIV indicator conditions in large hospitals can be time-consuming. Automating this screening by sensitive large language models is a promising use of artificial intelligence. Fourth, patients tested outside the hospitals were not captured, giving a risk of underestimating the effect size, and neither were asymptomatic patients from risk groups by an indicator condition testing strategy. Opportunities may also lie here in using large language models to help physicians identify this group of people, which could become more cost-effective and cost-efficient than using test-all strategies for asymptomatic patients at risk of HIV not covered by indicator-condition based testing. Fifth, the study did not collect provider-specific barriers to HIV testing. Sixth, changes in care during the pre and postimplementation periods, such as COVID-19, may have influenced the testing rate. The comparable overall testing rates before and during the COVID-19 pandemic [31] and similar preimplementation rates in the hospitals supports a limited impact. Seventh, inclusion of those with a first presentation of pneumonia would increase work-load but would pick up additional cases of HIV. Last, HIV indicator condition-guided testing does not cover asymptomatic people with an undiagnosed HIV infection. However, test-all strategies are often not cost-effective in countries with a low undiagnosed HIV prevalence.

## Conclusion

Implementing HIV teams hospital-wide with a multiangle approach significantly and sustainably increase HIV indicator condition-guided testing in hospitals. HIV teams should be further explored as a method of ensuring adequate HIV testing.

## Acknowledgements

We would like to thank all colleagues who provided feedback on our implementation project and helped us specify HIV indicator conditions from their point of view. Furthermore, we would like to thank our research master students for their help with data collection and outreach projects. This (preliminary) data was presented at the following conferences: CROI, EACS, HIV Glasgow, NCHIV, and NVHB.

Author contributions: Conceptualization C.J., J.S., J.K., A.V., C.R.; Data curation C.J.; Formal analysis; C.J.; Investigation; C.J., K.V., K.O., J.H., J.B., M.V., N.H., W.D., D.S.; Methodology; C.J., K.O., J.S., J.K., A.V., C.R.; Project administration; C.J.; Validation; C.J.; Visualization: C.J.; Writing – original draft C.J.; Supervision C.R.; Writing – review & editing all authors. All authors have read and approved the final version of the manuscript.

Disclosures: C.J. received travel funding from Gilead Sciences and received speaker funding from ViiV Healthcare, both outside the submitted work. C.R. received funding for investigator-initiated studies, and reimbursement for travel and participation in scientific advisory boards, from Gilead Sciences and ViiV Healthcare.

Funding: This study was supported by the Dutch Federation Medical Specialist (SKMS) (grant number: 59825822) and by an unrestricted investigator-initiated study grant from Gilead Sciences and ViiV Healthcare. The industry were not involved in the study design, data collection, analysis, interpretation, or submission to publish.

Trial registration: ClinicalTrials.gov NCT05225493.

### Conflicts of interest

There are no conflicts of interest.

## Supplementary Material

Supplemental Digital Content

## Supplementary Material

Supplemental Digital Content

## Supplementary Material

Supplemental Digital Content

## Data Availability

Data is available on request from the corresponding author.
