# Supplementary material for: Implementing HIV teams sustainably improves HIV indicator condition testing rates in hospitals in the Netherlands: the #aware.hiv clinical trial
Source: AIDS. 2025 Mar 18;39(8):995–1004. doi: 10.1097/QAD.0000000000004167 (PMC12144530; doi:10.1097/QAD.0000000000004167)
Supplement: Supplemental Digital Content [file aids-39-0995-s001.docx]

Supplementary data

**Figure 1: Implementation process at EMC and LUMC**


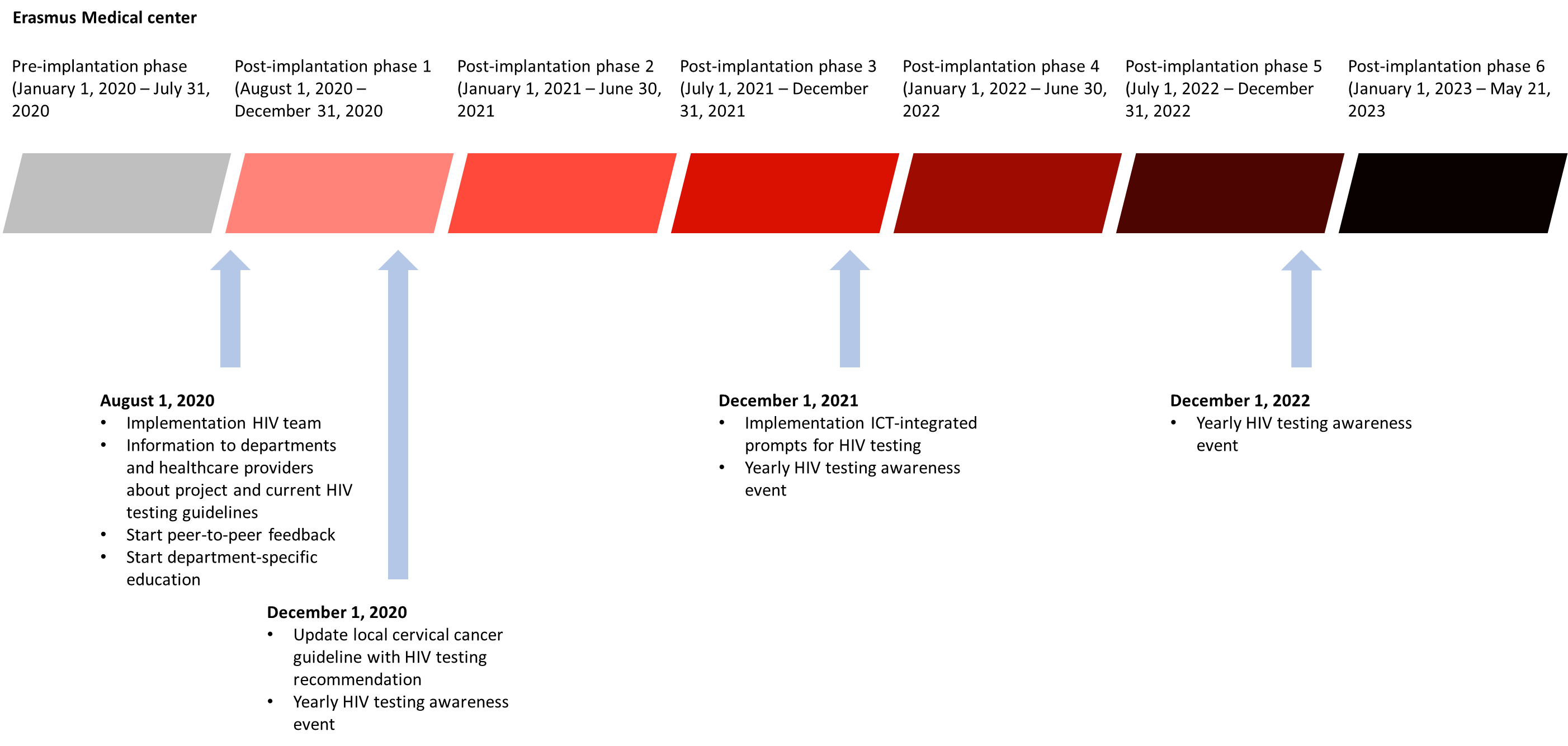


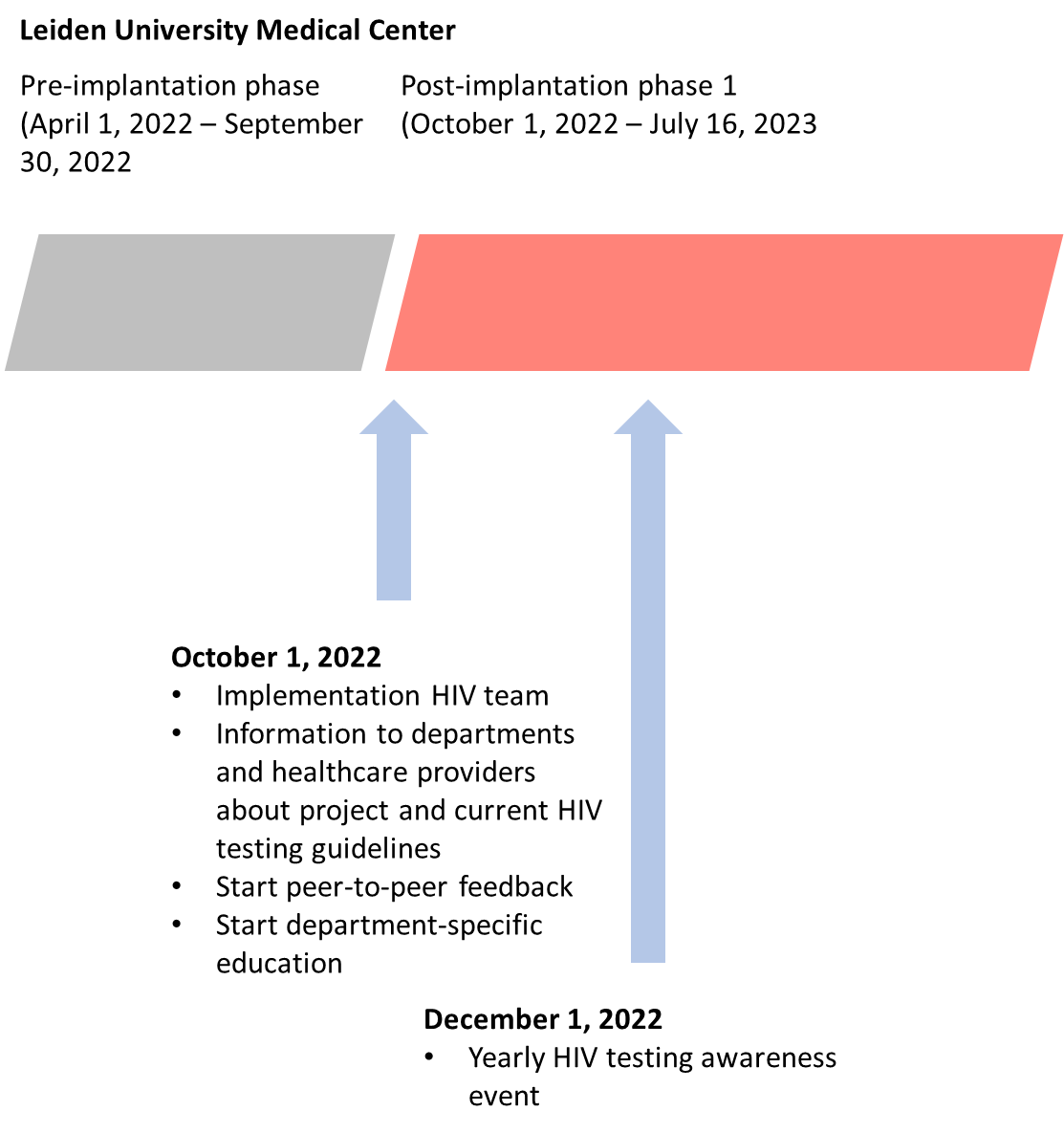


**Table 1: Prevalence and testing rate per HIV indicator condition, pre-implementation and post-implementation of HIV teams at EMC**

|  | **Prevalence of HIV indicator conditions** | | **HIV testing rate** | |
| --- | --- | --- | --- | --- |
| **HIV indicator condition** | **Pre-implementation of HIV teams, n (%)** | **Post-implementation of HIV teams, n (%)** | **Pre-implementation of HIV teams, n (%)** | **Post-implementation of HIV teams, n (%)** |
| **AIDS defining conditions** | | | | |
| **Neoplasms** | | | | |
| Cervical cancer | 44 (9.9) | 381 (19.5) | 0 (0.0) | 279 (73.2) |
| Non-Hodgkin lymphoma | 41 (9.3) | 272 (13.9) | 38 (92.7) | 261 (96.0) |
| Kaposi’s sarcoma | 6 (1.4) | 14 (0.7) | 4 (66.7) | 13 (92.9) |
| **Bacterial infections** | | | | |
| *Mycobacterium tuberculosis* | 18 (4.1) | 80 (4.1) | 9 (50.0) | 66 (82.5) |
| *Mycobacterium Kansasii* | - | 2 (0.1) | - | 1 (50.0) |
| *Mycobacterium*, other species | - | 1 (0.1) | - | 1 (100.0) |
| Pneumonia, recurrent | 4 (0.9) | 9 (0.5) | 2 (50.0) | 9 (100.0) |
| **Viral infections** | | | | |
| Progressive multifocal leucoencephalopathy | - | 1 (0.1) | - | 1 (100.0) |
| **Parasitic infections** | | | | |
| Cerebral toxoplasmosis | 2 (0.5) | 12 (0.6) | 0 (0.0) | 6 (50.0) |
| **Fungal infections** | | | | |
| Candidiasis, esophageal | - | 1 (0.1) | - | 0 (0.0) |
| Candidiasis | - | 3 (0.2) | - | 3 (100.0) |
| Histoplasmosis | - | 1 (0.1) | - | 1 (100.0) |
| **Conditions associated with an undiagnosed HIV prevalence >0.1%** | | | | |
| Sexually transmitted infections | 40 (9.0) | 193 (9.9) | 12 (30.0) | 121 (62.7) |
| Malignant lymphoma | 4 (0.9) | 67 (3.4) | 3 (75.0) | 66 (98.5) |
| Anal cancer | 9 (2.0) | 41 (2.1) | 7 (77.8) | 33 (80.5) |
| Herpes zoster | 11 (2.5) | 34 (1.7) | 0 (0.0) | 17 (50.0) |
| Hepatitis A | - | 3 (0.2) | - | 3 (100.0) |
| Hepatitis B | 33 (7.4) | 147 (7.5) | 27 (81.8) | 142 (96.6) |
| Hepatitis C | 15 (3.4) | 48 (2.5) | 12 (80.0) | 47 (97.9) |
| Mononucleosis-like illness | 5 (1.1) | 4 (0.2) | 1 (20.0) | 2 (50.0) |
| Unexplained leuko/thrombocytopenia | 14 (3.2) | 44 (2.3) | 11 (78.6) | 44 (100.0) |
| Seborrheic dermatitis/exanthema | 24 (5.4) | 11 (0.5) | 6 (25.0) | 5 (45.5) |
| Invasive pneumococcal disease | 10 (2.2) | 37 (1.9) | 3 (30.0) | 27 (73.0) |
| Unexplained fever | 14 (3.2) | 25 (1.3) | 13 (92.9) | 25 (100.0) |
| Primary lung cancer | 10 (2.2) | 13 (0.7) | 0 (0.0) | 1 (7.7) |
| Lymphocytic meningitis | 9 (2.0) | 50 (2.6) | 7 (77.8) | 47 (94.0) |
| Oral hairy leucoplakia | 1 (0.2) | - | 0 (0.0) | - |
| Severe or atypical psoriasis | 7 (1.6) | 77 (3.9) | 2 (28.6) | 28 (36.4) |
| Guillain-Barre syndrome | 6 (1.4) | 11 (0.5) | 2 (33.3) | 3 (27.3) |
| Mononeuritis | - | 4 (0.2) | - | 4 (100.0) |
| Subcortical dementia | 1 (0.2) | - | 0 (0.0) | - |
| Multiple sclerosis-like disease | 4 (0.9) | 9 (0.5) | 2 (50.0) | 9 (100.0) |
| Peripheral neuropathy | 24 (5.4) | 42 (2.2) | 4 (16.7) | 27 (64.3) |
| Unexplained weight loss | 15 (3.4) | 58 (3.0) | 13 (86.7) | 54 (93.1) |
| Unexplained lymphadenopathy | 15 (3.4) | 89 (4.6) | 13 (86.7) | 78 (87.6) |
| Unexplained oral candidiasis | 1 (0.2) | 5 (0.2) | 0 (0.0) | 4 (80.0) |
| Unexplained chronic diarrhea | 18 (4.1) | 12 (0.6) | 3 (16.7) | 8 (66.7) |
| Unexplained chronic renal impairment | 26 (5.9) | 68 (3.5) | 16 (61.5) | 65 (95.6) |
| **Conditions where not identifying the presence of an underlying HIV infection may have significant adverse implications for the individual clinical management** | | | | |
| Primary space occupying lesion of the bran | 9 (2.0) | 49 (2.5) | 9 (100.0) | 44 (89.8) |
| Idiopathic thrombocytopenic purpura | 3 (0.7) | 28 (1.4) | 3 (100.0) | 25 (92.6) |
| Thrombotic thrombocytopenic purpura | - | 7 (0.4) | - | 6 (85.7) |
| **Total** | **443** | **1,952** | **222 (50.1)** | **1,575 (80.7)** |

HIV = human immunodeficiency virus

**Supplementary table 2: testing rates per sexually transmitted infection at EMC, pre- and post-implementation of HIV teams**

|  | **Pre-implementation of HIV teams** | | | **Post-implementation of HIV teams** | | |
| --- | --- | --- | --- | --- | --- | --- |
| **Sexually transmitted infection** | **Tested for HIV, n (%)** | **Not tested for HIV, n (%)** | **Total** | **Tested for HIV, n (%)** | **Not tested for HIV, n (%)** | **Total** |
| Lymphogranuloma venereum | - | - | - | 1 (100.0) | 0 (0.0) | 1 |
| Sex accident | 2 (100.0) | 0 (0.0) | 2 | 1 (100.0) | 0 (0.0) | 1 |
| Syphilis | 4 (80.0) | 1 (20.0) | 5 | 42 (95.5) | 2 (4.5) | 44 |
| Genital herpes | 2 (33.3) | 4 (66.7) | 6 | 16 (53.3) | 14 (46.7) | 30 |
| Condyloma acuminata | 4 (15.4) | 22 (84.6) | 26 | 58 (52.7) | 52 (47.3) | 110 |
| Chlamydia Trachomatis | - | - | - | 2 (50.0) | 2 (50.0) | 4 |
| Gonorrhea | 0 (0.0) | 1 (100.0) | 1 | 1 (50.0) | 1 (50.0) | 2 |
| Trichomoniasis | - | - | - | 0 (0.0) | 1 (100.0) | 1 |
| **Total** | **12 (30.0)** | **28 (70.0)** | **40** | **121 (62.7)** | **72 (37.3)** | **193** |

**Supplementary table 3: HIV testing rates per HIV indicator condition per department at EMC after implementation of HIV teams**

| **HIV indicator condition** | **Tested for HIV,**  **n (%)** | **Not tested for HIV,**  **n (%)** | **Total** |
| --- | --- | --- | --- |
| **Dermatology/venereology** | | | |
| ***Mycobacterium tuberculosis*** | 2 (100.0) | 0 (0.0) | 2 |
| **Non-Hodgkin lymphoma** | 2 (100.0) | 0 (0.0) | 2 |
| **Anal cancer** | 1 (100.0) | 0 (0.0) | 1 |
| **Unexplained lymphadenopathy** | 1 (100.0) | 0 (0.0) | 1 |
| **Kaposi's sarcoma** | 6 (85.7) | 1 (14.3) | 7 |
| **Hepatitis B** | 2 (66.7) | 1 (33.3) | 3 |
| **Sexually transmitted infections** | 79 (58.5) | 56 (41.5) | 135 |
| **Herpes zoster** | 9 (50.0) | 9 (50.0) | 18 |
| **Seborrheic dermatitis/exanthema** | 5 (45.5) | 6 (54.5) | 11 |
| **Severe or atypical psoriasis** | 22 (35.5) | 40 (64.5) | 62 |
| **Unexplained oral candidiasis** | 0 (0.0) | 1 (100.0) | 1 |
| **Gynaecology/obstetrics** | | | |
| **Cervical cancer** | 173 (74.6) | 59 (25.4) | 232 |
| **Sexually transmitted infections** | 9 (52.9) | 8 (47.1) | 17 |
| **Pulmonology** | | | |
| **Non-Hodgkin lymphoma** | 7 (100.0) | 0 (0.0) | 7 |
| **Pneumonia** | 3 (100.0) | 0 (0.0) | 3 |
| **Malignant lymphoma** | 3 (100.0) | 0 (0.0) | 3 |
| **Hepatitis B** | 2 (100.0) | 0 (0.0) | 2 |
| **Unexplained weight loss** | 1 (100.0) | 0 (0.0) | 1 |
| **Unexplained thrombocytopenia/leukocytopenia** | 1 (100.0) | 0 (0.0) | 1 |
| **Candidiasis** | 1 (100.0) | 0 (0.0) | 1 |
| **Primary space occupying lesion of the brain** | 1 (100.0) | 0 (0.0) | 1 |
| ***Mycobacterium tuberculosis*** | 42 (89.4) | 5 (10.6) | 47 |
| **Invasive pneumococcal disease** | 12 (75.0) | 4 (25.0) | 16 |
| **Unexplained lymphadenopathy** | 13 (72.2) | 5 (27.8) | 18 |
| ***Mycobacterium kansasii*** | 1 (50.0) | 1 (50.0) | 2 |
| **Lung cancer** | 0 (0.0) | 8 (100.0) | 8 |
| **Herpes zoster** | 0 (0.0) | 1 (100.0) | 1 |
| **Candidiasis, esophageal** | 0 (0.0) | 1 (100.0) | 1 |
| **Neurology/neurosurgery** | | | |
| **Multiple sclerosis-like disease** | 9 (100.0) | 0 (0.0) | 9 |
| **Malignant lymphoma** | 5 (100.0) | 0 (0.0) | 5 |
| **Mononeuritis** | 4 (100.0) | 0 (0.0) | 4 |
| **Cervical cancer** | 1 (100.0) | 0 (0.0) | 1 |
| **Hepatitis C** | 1 (100.0) | 0 (0.0) | 1 |
| **Progressive multifocal leukoencephalopathy** | 1 (100.0) | 0 (0.0) | 1 |
| **Non-Hodgkin lymphoma** | 71 (96.0) | 3 (4.0) | 74 |
| **Lymphocytic meningitis** | 45 (93.8) | 3 (6.2) | 48 |
| **Primary space occupying lesion of the brain** | 18 (90.0) | 2 (10.0) | 20 |
| **Sexually transmitted infections** | 7 (87.5) | 1 (12.5) | 8 |
| ***Mycobacterium tuberculosis*** | 3 (75.0) | 1 (25.0) | 4 |
| **Herpes zoster** | 2 (66.7) | 1 (33.3) | 3 |
| **Polyneuropathy** | 25 (62.5) | 15 (37.5) | 40 |
| **Invasive pneumococcal disease** | 1 (50.0) | 1 (50.0) | 2 |
| **Guillain-Barre syndrome** | 3 (27.3) | 8 (72.7) | 11 |
| **Internal Medicine** | | | |
| **Unexplained thrombocytopenia/leukocytopenia** | 41 (100.0) | 0 (0.0) | 41 |
| **Unexplained fever** | 24 (100.0) | 0 (0.0) | 24 |
| **Primary space occupying lesion of the brain** | 14 (100.0) | 0 (0.0) | 14 |
| **Pneumonia** | 6 (100.0) | 0 (0.0) | 6 |
| **Kaposi's sarcoma** | 5 (100.0) | 0 (0.0) | 5 |
| **Hepatitis B** | 4 (100.0) | 0 (0.0) | 4 |
| **Unexplained oral candidiasis** | 2 (100.0) | 0 (0.0) | 2 |
| **Hepatitis C** | 2 (100.0) | 0 (0.0) | 2 |
| **Polyneuropathy** | 1 (100.0) | 0 (0.0) | 1 |
| **Histoplasmosis** | 1 (100.0) | 0 (0.0) | 1 |
| **Candidiasis** | 1 (100.0) | 0 (0.0) | 1 |
| **Cerebral toxoplasmosis** | 1 (100.0) | 0 (0.0) | 1 |
| ***Mycobacterium,* other species** | 1 (100.0) | 0 (0.0) | 1 |
| **Malignant lymphoma** | 53 (98.1) | 1 (1.9) | 54 |
| **Non-Hodgkin lymphoma** | 163 (96.5) | 6 (3.5) | 169 |
| **Unexplained chronic renal impairment** | 51 (96.2) | 2 (3.8) | 53 |
| **Unexplained weight loss** | 53 (93.0) | 4 (7.0) | 57 |
| **Unexplained lymphadenopathy** | 48 (92.3) | 4 (7.7) | 52 |
| **Idiopathic thrombocytopenic purpura** | 25 (92.6) | 2 (7.4) | 27 |
| **Sexually transmitted infections** | 19 (90.5) | 2 (9.5) | 21 |
| **Thrombotic thrombocytopenic purpura** | 6 (85.7) | 1 (14.3) | 7 |
| **Anal cancer** | 28 (82.4) | 6 (17.6) | 34 |
| **Herpes zoster** | 4 (80.0) | 1 (20.0) | 5 |
| **Unexplained chronic diarrhea** | 4 (80.0) | 1 (20.0) | 5 |
| **Cervical cancer** | 105 (70.9) | 43 (29.1) | 148 |
| **Invasive pneumococcal disease** | 12 (70.6) | 5 (29.4) | 17 |
| ***Mycobacterium tuberculosis*** | 11(61.1) | 7 (38.9) | 18 |
| **Mononucleosis-like illness** | 1 (50.0) | 1 (50.0) | 2 |
| **Gastroenterology/hepatology** | | | |
| **Hepatitis A** | 3 (100.0) | 0 (0.0) | 3 |
| **Unexplained fever** | 1 (100.0) | 0 (0.0) | 1 |
| **Anal cancer** | 1 (100.0) | 0 (0.0) | 1 |
| **Unexplained lymphadenopathy** | 1 (100.0) | 0 (0.0) | 1 |
| **Unexplained thrombocytopenia/leukocytopenia** | 1 (100.0) | 0 (0.0) | 1 |
| ***Mycobacterium tuberculosis*** | 1 (100.0) | 0 (0.0) | 1 |
| **Sexually transmitted infections** | 1 (100.0) | 0 (0.0) | 1 |
| **Hepatitis C** | 44 (97.8) | 1 (2.2) | 45 |
| **Hepatitis B** | 132 (97.1) | 4 (2.9) | 136 |
| **Unexplained chronic diarrhea** | 3 (50.0) | 3 (50.0) | 6 |
| **Unexplained chronic renal impairment** | 1 (50.0) | 1 (50.0) | 2 |

HIV = human immunodeficiency virus

**Supplementary table 4:** **Reasons given not to test for HIV at EMC**

| **Reason not to test for HIV** | **n, (%)** |
| --- | --- |
| No reason provided | 173 (50.6) |
| Patient did not return to hospital after HIV test advice | 63 (18.4) |
| HIV test in diagnostic plan, but not performed | 35 (10.2) |
| Physician assumed there was no clinical indication to test for HIV | 29 (8.5) |
| Physician advised to perform HIV test somewhere else | 12 (3.5) |
| Patient was in palliative stage | 7 (2.0) |
| HIV test was ordered by physician, but not performed | 7 (2.0) |
| Shared decision making physician and patient to not perform HIV test | 5 (1.5) |
| HIV test offered by physician, but not accepted by patient | 4 (1.2) |
| Patient died soon after HIV test advice was given | 1 (0.3) |
| Physician forgot to test for HIV | 1 (0.3) |
| HIV test in diagnostic plan, patient refused to come back to the department | 1 (0.3) |
| Patient wants no further appointments | 1 (0.3) |
| Physician assumed that HIV testing was done elsewhere | 1 (0.3) |
| Physician thinks that other specialty should test for HIV | 1 (0.3) |
| Physician reports that patient has been recently tested elsewhere, but no documentation on HIV test result has been documented | 1 (0.3) |
| **Total** | **342** |

HIV = human immunodeficiency virus

**Supplementary table 5: Testing rates after HIV testing advice was given and reasons not to test for HIV at EMC**

| **HIV indicator condition** | **Tested for HIV, n (%)** | **Not tested for HIV, n (%)** | **Reason not to test** |
| --- | --- | --- | --- |
| **AIDS defining conditions** | | | |
| **Neoplasms** | | | |
| Cervical cancer | 15 (13.4) | 97 (86.6) | No reason provided (n=57) HIV test in diagnostic plan but not performed (n=24)  Patient did not return to hospital after HIV test advice (n=6) HIV test was ordered by physician but not performed (n=4) Patient was in palliative stage (n=4) Physician assumed there was no clinical indication to test for HIV (n=2) |
| Non-Hodgkin lymphoma | 6 (37.5) | 10 (62.5) | No reason provided (n=4) Patient did not return to hospital after HIV test advice (n=3) HIV test was ordered by physician but not performed (n=1) Patient was in palliative stage (n=1) Physician assumed there was no clinical indication to test for HIV (n=1) |
| Kaposi's sarcoma | 0 (0.0) | 1 (100.0) | No reason provided (n=1) |
| **Bacterial infections** | | | |
| *Mycobacterium tuberculosis* | 3 (17.6) | 14 (82.4) | No reason provided (n=8) Physician assumed there was no clinical indication to test for HIV (n=2)  Patient did not return to hospital after HIV test advice (n=1) HIV test in diagnostic plan but not performed (n=1)  Patient died soon after HIV test advice was given (n=1)  Patient wants no further appointments (n=1) |
| *Mycobacterium kansasii* | 0 (0.0) | 1 (100.0) | Patient did not return to hospital after HIV test advice (n=1) |
| **Parasitic infections** | | | |
| Cerebral toxoplasmosis | 0 (0.0) | 6 (100.0) | No reason provided (n=3)  Patient did not return to hospital after HIV test advice (n=3) |
| **Fungal infections** | | | |
| Candidiasis, esophageal | 0 (0.0) | 1 (100.0) | Physician advised to perform HIV test somewhere else (n=1) |
| **Conditions associated with an undiagnosed HIV prevalence >0.1%** | | | |
| Sexually transmitted infections | 16 (19.5) | 66 (80.5) | No reason provided (n=30) Patient did not return to hospital after HIV test advice (n=15)  Physician advised to perform HIV test somewhere else (n=11)  HIV test in diagnostic plan but not performed (n=3) Physician assumed there was no clinical indication to test for HIV (n=3) HIV testing in diagnostic plan, but patient refused to come back to the department (n=1) HIV test offered by physician but not accepted by patient (n=1) Physician thinks other specialty should test for HIV (n=1) Physician reports that patient has been recently tested elsewhere, but no documentation on HIV test result has been documented (n=1) |
| Malignant lymphoma | 0 (0.0) | 1 (100.0) | Physician forgot to test for HIV (n=1) |
| Anal cancer | 3 (30.0) | 7 (70.0) | No reason provided (n=3)  HIV test in diagnostic plan but not performed (n=1)  Patient was in palliative stage (n=1)  Physician assumed there was no clinical indication to test for HIV (n=1)  Shared decision making physician and patient to not perform HIV test (n=1) |
| Herpes zoster | 0 (0.0) | 12 (100.0) | No reason provided (n=8)  Patient did not return to hospital after HIV test advice (n=4) |
| Hepatitis B | 5 (50.0) | 5 (50.0) | No reason provided (n=1)  Patient did not return to hospital after HIV test advice (n=3)  HIV test in diagnostic plan but not performed (n=1) |
| Hepatitis C | 2 (66.7) | 1 (33.3) | Patient did not return to hospital after HIV test advice (n=1) |
| Mononucleosis-like illness | 0 (0.0) | 2 (100.0) | Patient did not return to hospital after HIV test advice (n=1)  Physician assumed there was no clinical indication to test for HIV (n=1) |
| Seborrheic dermatitis/exanthema | 1 (14.3) | 6 (85.7) | No reason provided (n=3)  HIV test in diagnostic plan but not performed (n=2) Patient did not return to hospital after HIV test advice (n=1) |
| Invasive pneumococcal disease | 3 (23.1) | 10 (76.9) | Patient did not return to hospital after HIV test advice (n=8) Patient was in palliative stage (n=1)  Patient did not return to hospital after HIV test advice (n=1) |
| Lung cancer | 0 (0.0) | 9 (100.0) | No reason provided (n=4)  Physician assumed there was no clinical indication to test for HIV (n=2)  Patient did not return to hospital after HIV test advice (n=1)  HIV test in diagnostic plan but not performed (n=1)  HIV test was ordered by physician but not performed (n=1) |
| Lymphocytic meningitis | 2 (50.0) | 2 (50.0) | No reason provided (n=1)  Physician assumed there was no clinical indication to test for HIV (n=1) |
| Psoriasis | 7 (12.7) | 48 (87.3) | No reason provided (n=31) Physician assumed there was no clinical indication to test for HIV (n=6)  Patient did not return to hospital after HIV test advice (n=4) Shared decision making physician and patient to not perform HIV test (n=3)  HIV test in diagnostic plan but not performed (n=2) HIV test offered by physician but not accepted by patient (n=1)  HIV test was ordered by physician but not performed (n=1) |
| Guillain-Barre syndrome | 0 (0.0) | 8 (100.0) | No reason provided (n=5)  Patient did not return to hospital after HIV test advice (n=2) Physician assumed there was no clinical indication to test for HIV (n=1) |
| Peripheral neuropathy | 1 (10.0) | 9 (90.0) | No reason provided (n=4)  Physician assumed there was no clinical indication to test for HIV (n=3)  Patient did not return to hospital after HIV test advice (n=2) |
| Unexplained weight loss | 1 (25.0) | 3 (75.0) | No reason provided (n=1)  Patient did not return to hospital after HIV test advice (n=1)  Shared decision making physician and patient to not perform HIV test (n=1) |
| Unexplained lymphadenopathy | 1 (9.1) | 10 (90.9) | No reason provided (n=6)  Patient did not return to hospital after HIV test advice (n=2)  Physician assumed there was no clinical indication to test for HIV (n=2) |
| Unexplained oral candidiasis | 0 (0.0) | 1 (100.0) | Patient did not return to hospital after HIV test advice (n=1) |
| Unexplained chronic diarrhea | 0 (0.0) | 3 (100.0) | No reason provided (n=1)  HIV test offered by physician but not accepted by patient (n=1)  Physician assumed there was no clinical indication to test for HIV (n=1) |
| Unexplained chronic renal impairment | 0 (0.0) | 2 (100.0) | No reason provided (n=1)  Physician assumed there was no clinical indication to test for HIV (n=1) |
| **Conditions where not identifying the presence of an underlying HIV infection may have significant adverse implications for the individual clinical management** | | | |
| Primary space occupying lesion of the brain | 2 (28.6) | 5 (71.4) | Patient did not return to hospital after HIV test advice (n=3)  Physician assumed there was no clinical indication to test for HIV (n=2) |
| ITP/TTP | 1 (33.3) | 2 (66.7) | No reason provided (n=1)  Physician assumed that HIV testing was done elsewhere (n=1) |

HIV = human immunodeficiency virus, ITP = idiopathic thrombocytopenic purpura, TTP = Thrombotic thrombocytopenic purpura

**Supplementary table 6: Testing rate per HIV indicator condition before and after implementation of ICT-integrated diagnostics at EMC**

|  | **Pre-implementation* ICT-integrated diagnostics** | | | **Post-implementation** ICT-integrated diagnostics** | | |
| --- | --- | --- | --- | --- | --- | --- |
| **Indicator condition** | **Tested for HIV, n (%)** | **Not tested for HIV, n (%)** | **Total** | **Tested for HIV, n (%)** | **Not tested for HIV, n (%)** | **Total** |
| **Cryptococcosis** | - | - | - | 1 (100.0) | 0 (0.0) | 1 |
| **Mycobacterium tuberculosis** | 27 (93.1) | 2 (6.9) | 29 | 17 (94.4) | 1 (5.6) | 18 |
| **Syphilis** | 21 (87.5) | 3 (12.5) | 24 | 24 (92.3) | 2 (7.7) | 26 |
| **Gonorrhea** | 13 (65.0) | 7 (35.0) | 20 | 5 (71.4) | 2 (28.6) | 7 |
| **Invasive pneumococcal disease** | 25 (39.1) | 39 (60.9) | 64 | 51 (70.8) | 21 (29.2) | 72 |
| **Chlamydia** | 30 (53.6) | 26 (46.4) | 56 | 17 (54.8) | 14 (45.6) | 31 |
| **Toxoplasmosis** | 3 (60.0) | 2 (40.0) | 5 | 1 (50.0) | 1 (50.0) | 2 |
| **Mycobacterium avium** | 1 (100.0) | 0 (0.0) | 1 | 0 (0.0) | 1 (100.0) | 1 |
| **Total** | 120 (60.3) | 79 (39.7) | 199 | 116 (73.4) | 42 (26.6) | 158 |

* August 2020 – August 2022, HIV team was already implemented
** December 2022 – December 2023

**Supplementary figure 2: flowchart LUMC**


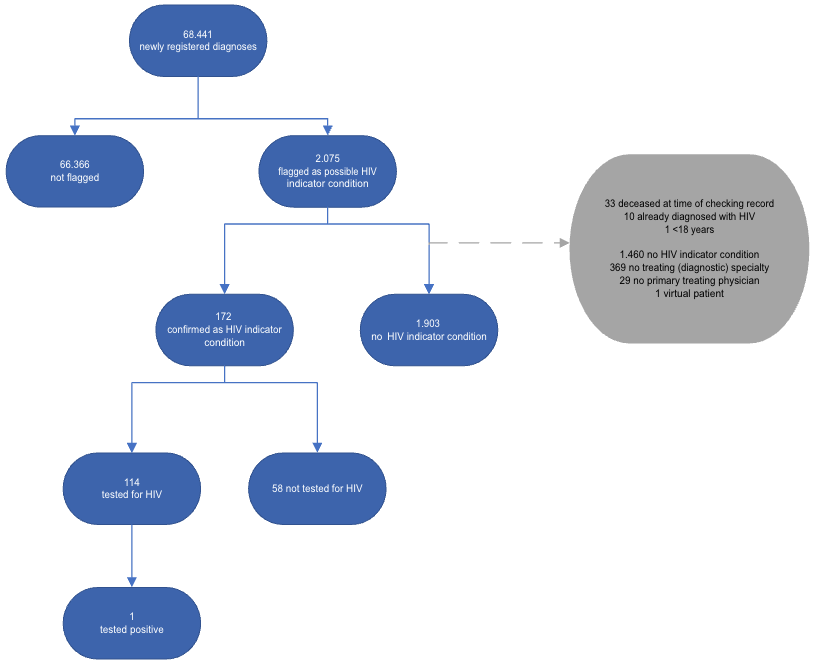


HIV = human immunodeficiency virus
