# Supplementary material for: Implementing HIV teams sustainably improves HIV indicator condition testing rates in hospitals in the Netherlands: the #aware.hiv clinical trial
Source: AIDS. 2025 Mar 18;39(8):995–1004. doi: 10.1097/QAD.0000000000004167 (PMC12144530; doi:10.1097/QAD.0000000000004167)
Supplement: Supplemental Digital Content [file aids-39-0995-s002.docx]

**Appendix A: Full list of HIV indicator conditions including their clinical definition**

| **HIV indicator condition** | **Clinical definition** | **Excluded if*:** |
| --- | --- | --- |
| **AIDS defining conditions** | | |
| Cervical cancer | All cases of cervical cancer, independent of HPV |  |
| Non-Hodgkin Lymphoma | All cases of the following lymphomas:  Diffuse Large B-cell Lymphoma (DLBCL)  Hodgkin lymphoma  Castleman lymphoma  Primary effusion lymphoma  Aggressive lymphoma (high grade B-cell lymphoma) | Mantle Cell lymphoma  T-cell Non-Hodgkin lymphoma (NHL)  Follicular lymphoma  Marginal zone lymphoma  MALT lymphoma  Post-transplant lymphoma (PTLD)  Exclude all lymphoma’s related to congenital defects/syndromes |
| Kaposi’s sarcoma | All cases of Kaposi sarcoma |  |
| Mycobacterium tuberculosis, pulmonary or extrapulmonary | All cases of active tuberculosis, or if treatment was initiated without positive sputum sample  Latent tuberculosis only when other risk factors for HIV are present |  |
| Mycobacterium avium complex or Mycobacterium kansasii, disseminated or extrapulmonary | All disseminated or extrapulmonary cases of mycobacterium Avium or mycobacterium kansasii |  |
| Pneumonia, recurrent (2 or more episodes in 12 months) | At least 2 episodes of pneumonia in a 12 month time period  Pneumonia can be diagnosed clinically on 2 separate occasions, through detected pathogens, CRP levels, and X-thorax showing 2 different infiltrates | Influenza A COVID  Viral infection  Aspiration pneumonia  Obstruction pneumonia  Organizing pneumonia |
| Cytomegalovirus retinitis | All cases of cytomegalovirus retinitis |  |
| Cytomegalovirus, other (except liver, spleen, glands) | All cases of cytomegalovirus, except liver, spleen, glands |  |
| Herpes simplex ulcer(s) >1 month / bronchitis / pneumonitis | All cases of herpes simplex ulcer(s) lasting >1 month  All cases of herpes simplex bronchitis or pneumonitis |  |
| Cerebral toxoplasmosis | Cerebral toxoplasmosis  Retinal toxoplasmosis |  |
| Cryptosporidiosis diarrhoea, >1 month | All cases of cryptosporidiosis diarrhoea lasting >1 month |  |
| Isosporiasis, >1 month | All cases of isosporiasis diarrhoea lasting >1 month |  |
| Atypical disseminated leishmaniasis | All cases of atypical disseminated visceral leishmaniasis |  |
| Reactivation of American trypanosomiasis (meningoencephalitis or myocarditis) | All cases of reactivation of American trypanosomiasis |  |
| Pneumocystis carinii pneumonia (PCP/PJP) | All cases of pneumocystis carinii pneumonia |  |
| Candidiasis, oesophageal | All cases of unexplained oesophageal candidiasis | Explained if Poorly regulated diabetes  Use of proton pump inhibitors  Use of inhalation corticosteroids  Stenose or ulcer in oesophagus  Radiotherapy or resection of the oesophagus |
| Candidiasis, bronchial / tracheal / lungs | All cases of unexplained bronchial, tracheal or lung candidiasis | Explained if Poorly regulated diabetes |
| Cryptococcosis, extra-pulmonary | All cases of extra-pulmonary cryptococcosis |  |
| Histoplasmosis, disseminated / extra-pulmonary | All cases of disseminated or extra-pulmonary histoplasmosis |  |
| Coccidiomycosis, disseminated / extrapulmonary | All cases of disseminated or extra-pulmonary coccidiomycosis |  |
| Penicilliosis, disseminated | All cases of disseminated penicilliosis |  |
| **Conditions with an undiagnosed HIV prevalence >0.1%** | | |
| Sexually transmitted infections | All new cases of STIs require testing in that disease episode  Symptomatically approached/treated STIs  Pelvic inflammatory disease (PID) caused by an STI  Condylomata  Scabies with crustae | Normal scabies  Ureaplasma |
| Malignant lymphoma | All cases of malignant lymphoma |  |
| Anal cancer / dysplasia | All cases of anal cancer or dysplasia  Independent of HPV |  |
| Cervical dysplasia | All cases of cervical dysplasia with PAP3a or CIN I  Independent of HPV | PAP 0 (not enough cells)  PAP 1 (good result)  PAP 2 (possibly self-limiting) |
| Herpes zoster | All cases of herpes zoster, regardless of age  Also if presenting with postherpetic neuralgy or secondary infection | Primo infection (varicella zoster) |
| Hepatitis A | All new cases of hepatitis A |  |
| Hepatitis B (acute or chronic) | All cases of hepatitis B regardless of chronicity and disease activity  Chronic hepatitis B needs to be tested at least once since diagnosis |  |
| Hepatitis C (acute or chronic) | All cases of hepatitis C regardless of chronicity and disease activity  Chronic hepatitis C needs to be tested at least once since diagnosis |  |
| Mononucleosis-like illness | Acute tonsillitis with negative EBV test Tonsillitis with longstanding fever Tonsillitis with insufficient response on antibiotics | Excluded if: serologically or molecularly proven recent EBV or CMV infection, chronic tonsillitis, or other diagnosed viral illness associated with a flu-like fever |
| Unexplained thrombocytopenia/leukocytopenia lasting >4 weeks | Include when thrombo/leukocytopenia is present after 4 weeks and no cause has been found after laboratory tests (including autoimmune tests) and ultrasound of the abdomen |  |
| Seborrheic dermatitis / exanthema | Extensive or difficult to treat seborrheic dermatitis / exanthema  Impetiginized seborrheic dermatitis / exanthema | Atopic eczema  Acrovesiculous / dyshidrotic eczema  Eczema hyperkeratoticum  Asteatotic eczema  Hypostatic eczema  Eczema of vulva / perineal area |
| Invasive pneumococcal disease | All cases of invasive pneumococcal disease, meaning pneumococci are detected in extra pulmonary material and sterile material, including a positive antigen test in the urine |  |
| Unexplained fever | Unexplained fever, lasting longer than 4 weeks | Fever with known pathogenesis |
| Candidaemia | All cases of unexplained candidaemia | Explained if Poorly regulated diabetes |
| Visceral leishmaniasis | All cases of visceral leishmaniasis |  |
| Primary lung cancer | Only cases of primary lung cancer when they also have at least one risk factor for HIV |  |
| Lymphocytic meningitis | Unexplained mononuclear pleocytosis in the CSF (leukocytes ≥5 mm^3^ AND >50% MNC) | Auto-immune encephalitis |
| Oral hairy leukoplakia | All cases of unexplained oral hairy leukoplakie |  |
| Severe or atypical psoriasis | All severe or atypical forms of psoriasis, including  New, fast progressing psoriasis  Psoriasis with erythrodermia  Severe psoriasis with reactive arthritis  Combination of seborrheic eczema and psoriasis (sebopsoriasis) Impetiginized psoriasis  Combination of different phenotypes of psoriasis | Easy to treat, typical psoriasis  Reactive arthritis with mild psoriasis |
| Guillain-Barré syndrome | All cases of unexplained Guillan-Barré syndrome or prior episode of Guillain-Barré syndrome |  |
| Mononeuritis | Unexplained mononeuritis which is rapidly progressive and painful and has been confirmed through EMG or neurological examination |  |
| Subcortical dementia | All cases of subcortical dementia |  |
| Multiple sclerosis-like disease | All cases of unexplained MS-like disease or in case neurologists assume MS which is insufficiently supported by findings in the diagnostic work up | Diagnosed MS |
| Peripheral neuropathy | All cases of unexplained peripheral neuropathy  Often symmetrical and feet more affected than hands  Neuropathy has been confirmed through EMG or during neurological examination | Explained if: (Poorly regulated) diabetes  Neuropathic pain after operation  Pressure neuropathy  Iatrogenic nerve injury  Traumatic nerve injury  Nitrous oxide use or vitamin B12 deficiency  MGUS  ACNES  Neuroma  Post chemotherapy |
| Unexplained weight loss | At least 5% weight loss in 6 months,  No explanation despite one round of diagnostics (for example CT or colonoscopy) |  |
| Unexplained lymphadenopathy | Include when lymphadenopathy is present and no cause has been found after laboratory tests and punction / excision of lymph node |  |
| Unexplained oral candidiasis | All cases of unexplained oral candida | Explained if Poorly regulated diabetes  Use of proton pump inhibitors  Use of inhalation corticosteroids |
| Unexplained chronic diarrhea | Unexplained diarrhea lasting >4 weeks  Diarrhea caused by cryptosporidiosis, microsporidiosis or isosporidiosis | Pancolitis ulcera  Colitis  Viral gastroenteritis |
| Unexplained chronic renal impairment | All cases of undiagnosed chronic renal impairment lasting longer than 3 months, no explanation despite laboratory tests AND urine diagnostics AND ultrasound or biopsy  Focal segmental glomerulosclerosis | Post renal obstruction  IgA nephropathy  Diabetic nephropathy  Morbus Behcet  Venous occlusion  Post chemotherapy  aHUS  Hypertension  TIN-U |
| Candidiasis | All cases of unexplained oral candidiasis |  |
| **Conditions where not identifying the presence of HIV infection may have significant adverse implications** | | |
| Primary space occupying lesion of the brain | Abces in brain with unknown pathogen  Lymphoma (non-Hodgkin or malignant) of central nerve system | Glioblastoma  Meningioma  Astrocytoma  Metastasis in brain  Ependymoma  Schwannoma  Neuroma  Oligodendroglioma |
| Idiopathic thrombocytopenic purpura | All cases of idiopathic thrombocytopenic purpura |  |
| Thrombotic thrombocytopenic purpura | All cases of thrombotic thrombocytopenic purpura |  |

* Immunocompromised patients are always excluded as HIV indicator condition
