# Supplementary material for: Implementing HIV teams sustainably improves HIV indicator condition testing rates in hospitals in the Netherlands: the #aware.hiv clinical trial
Source: AIDS. 2025 Mar 18;39(8):995–1004. doi: 10.1097/QAD.0000000000004167 (PMC12144530; doi:10.1097/QAD.0000000000004167)
Supplement: Supplemental Digital Content [file aids-39-0995-s003.docx]

**Appendix B: full list of HIV indicator conditions included in ICT-integrated prompts for HIV testing**

| **Indicator condition** | **Test** | **Clinical samples** |
| --- | --- | --- |
| *Mycobacterium tuberculosis, (extra) pulmonary* | PCR  Culture + MALDI-TOF identification | All samples |
| *Mycobacterium avium* complex, pulmonary | PCR sequence typing  Culture + MALDI-TOF identification | All samples |
| *Salmonella* species septicemia | Culture + MALDI-TOF identification | Blood culture |
| Invasive pneumococcal disease  (*Streptococcus pneumoniae*) | Rapid pneumococcal test  Culture + MALDI-TOF identification | Urine  CSF  Blood culture |
| *Pneumocystis jirovecii* pneumonia | PCR | Respiratory fluid |
| Cerebral toxoplasmosis  (*Toxoplasma gondii*) | PCR  Serology (Toxoplasma differential agglutination test) serum/ CSF index | Brain tissue  Serum  CSF |
| Cryptococcosis,  extrapulmonary  (*Cryptococcus* species) | Antigen test  Culture + MALDI-TOF identification | Serum  CSF  Ascites  Blood culture |
| Chlamydia/ Lymphogranuloma venereum  (*Chlamydia trachomatis*) | PCR | All samples |
| Gonorrhoea  (*Neisseria gonorrhoeae)* | PCR  Culture + MALDI-TOF identification | All samples |
| Syphilis (*Treponema pallidum*) | PCR  VDRL ≥1:8 + IgG Immuno-Blot positive | All samples  Serum |

MALDI-TOF: Matrix assisted laser desorption/ionisation time-of-flight analyzer; CSF: cerebrospinal fluid; VDRL: Venereal disease research laboratory
